# Intact Proviral DNA Analysis of the Brain Viral Reservoir and Relationship to Neuroinflammation in People with HIV on Suppressive Antiretroviral Therapy

**DOI:** 10.3390/v15041009

**Published:** 2023-04-20

**Authors:** Dana Gabuzda, Jun Yin, Vikas Misra, Sukrutha Chettimada, Benjamin B. Gelman

**Affiliations:** 1 Department of Cancer Immunology and Virology, Dana-Farber Cancer Institute, Boston, MA 02215, USA; 2 Department of Neurology, Harvard Medical School, Boston, MA 02115, USA; 3 Department of Pathology, University of Texas Medical Branch, Galveston, TX 77555, USA

**Keywords:** HIV reservoirs, intact proviral DNA assay, intact proviral genomes, HIV-associated neurocognitive disorders, neuroinflammation

## Abstract

HIV establishes a persistent viral reservoir in the brain despite viral suppression in blood to undetectable levels on antiretroviral therapy (ART). The brain viral reservoir in virally suppressed HIV+ individuals is not well-characterized. In this study, intact, defective, and total HIV proviral genomes were measured in frontal lobe white matter from 28 virally suppressed individuals on ART using the intact proviral DNA assay (IPDA). HIV gag DNA/RNA levels were measured using single-copy assays and expression of 78 genes related to inflammation and white matter integrity was measured using the NanoString platform. Intact proviral DNA was detected in brain tissues of 18 of 28 (64%) individuals on suppressive ART. The median proviral genome copy numbers in brain tissue as measured by the IPDA were: intact, 10 (IQR 1–92); 3′ defective, 509 (225–858); 5′ defective, 519 (273–906); and total proviruses, 1063 (501–2074) copies/10^6^ cells. Intact proviral genomes accounted for less than 10% (median 8.3%) of total proviral genomes in the brain, while 3′ and 5′ defective genomes accounted for 44% and 49%, respectively. There was no significant difference in median copy number of intact, defective, or total proviruses between groups stratified by neurocognitive impairment (NCI) vs. no NCI. In contrast, there was an increasing trend in intact proviruses in brains with vs. without neuroinflammatory pathology (56 vs. 5 copies/10^6^ cells, *p* = 0.1), but no significant differences in defective or total proviruses. Genes related to inflammation, stress responses, and white matter integrity were differentially expressed in brain tissues with >5 vs. +5 intact proviruses/10^6^ cells. These findings suggest that intact HIV proviral genomes persist in the brain at levels comparable to those reported in blood and lymphoid tissues and increase CNS inflammation/immune activation despite suppressive ART, indicating the importance of targeting the CNS reservoir to achieve HIV cure.

## 1. Introduction

HIV establishes a persistent reservoir of infected cells in the brain despite viral suppression in blood to undetectable levels on combination antiretroviral therapy (ART) [1,2,3,4]. HIV enters the brain within weeks of acute infection, and early initiation of ART decreases the size of the CNS viral reservoir [5]. In virally suppressed HIV+ individuals on ART, most of the infected cells in the brain are myeloid cells (macrophages and microglia); evidence of latent or low-level productive infection persisting in these myeloid cell reservoirs includes studies using single-copy PCR assays, in situ detection of HIV RNA/DNA, and quantitative viral outgrowth assays (QVOA) [6,7,8,9,10,11]. CD4+ T-cells trafficking into the CNS are another reservoir of replication-competent HIV in the brain [4,12,13,14], while astrocytes may harbor a low-level infection [15].

HIV DNA and RNA is detected in the brain in the majority of HIV+ individuals examined at autopsy, including those with viral suppression in blood [1,7,10,11,16,17,18]. HIV RNA and DNA levels are generally higher in white matter than gray matter, and variable across neuroanatomical regions [1,6,10,15,17]. Although suppressive ART reduces plasma and CSF HIV RNA levels to below the limits of detection in most people, higher HIV RNA levels in CSF compared with plasma are observed in a minority of ART-treated individuals, a discordance referred to as CSF viral escape [19,20,21]. These findings highlight the importance of viral reservoirs in the brain as an obstacle to achieving HIV cure.

Neurocognitive impairment (NCI) remains prevalent among people with HIV despite suppressive ART [22,23]. Mechanisms underlying HIV-associated neurocognitive disorders (HAND), which include asymptomatic neurocognitive impairment (ANI), minor neurocognitive disorder (MND), and HIV-associated dementia (HAD), in ART-treated individuals are multifactorial and include ongoing low-level viral replication in the brain, neuroinflammation, ART neurotoxicity, cerebrovascular disease, and other factors [2,22,23,24,25]. Although HIV encephalitis (HIVE) is common in untreated HIV+ individuals, it is uncommon in virally suppressed individuals. Common neuropathological findings in virally suppressed individuals include mild nonspecific changes such as reactive gliosis, lymphocytic infiltrates, decreased white matter integrity, and cerebrovascular disease [11,16,22,26,27]. Previous studies identified gene expression changes in the brains of untreated and ART-treated HIV+ individuals, including upregulation of genes related to inflammation, interferon responses, stress responses, lipid/energy metabolism, and white matter integrity [11,28,29,30,31]. However, the contribution of intact proviral genomes to gene expression changes and NCI in virally suppressed individuals remains unclear.

The intact proviral DNA assay (IPDA) is a sensitive multiplex digital droplet PCR method that was recently developed to quantify intact and defective HIV proviral genomes in cells and tissues and estimate the size of the replication-competent proviral reservoir [32,33,34,35]. The IPDA has been validated in published studies which showed correlation of intact proviral DNA genome copies with other measures of replication-competent HIV proviruses such as quantitative viral outgrowth assays and detection of integrated HIV genomes [32,36,37]. IPDA analysis of CD4+ T-cell reservoirs in blood and lymphoid tissues shows that intact proviral genomes are detected in these compartments and the ratio of intact-to-total proviral genomes range is ~1–12% [32,36,37,38,39]. These studies of intact proviruses are important for evaluating effects of interventions on the size of the replication-competent viral reservoir and HIV cure.

The brain viral reservoir in virally suppressed HIV+ individuals is not well-characterized. In a recent study, Cochrane et al. measured intact and defective HIV proviral genomes in the brain tissue of 18 viremic and 12 virologically suppressed HIV+ individuals examined at autopsy [18]. Intact proviral genomes were detected in 8 of 10 viremic and 6 of 9 suppressed individuals, with no significant difference between levels of intact or total proviral DNA in untreated vs. virally suppressed individuals. CNS myeloid cells were shown to harbor HIV DNA in virally suppressed individuals. Here, we characterize intact, defective, and total HIV proviral genomes in frontal white matter tissue from 28 virally suppressed HIV+ individuals on ART and examine relationships between intact proviral genomes, neuroinflammation, and neurocognitive impairment.

## 2. Methods

### 2.1. Cohort Selection and Characterization

HIV+ individuals and age/sex-matched HIV-negative controls with available frozen autopsy brain tissue collected between 2001 and 2014 were from the National NeuroAIDS Tissue Consortium (NNTC) sites (Texas NeuroAIDS Research Center, National Neurological AIDS Bank, Manhattan HIV Brain Bank, and California NeuroAIDS Tissue Network) [40]. All subjects were enrolled with written informed consent and Institutional Review Board (IRB) approval at each study site. Autopsy tissue samples and clinical data were collected and coded to protect participants’ confidentiality in accordance with IRB approved protocols at the University of Texas Medical Branch Galveston, University of California Los Angeles, Icahn School of Medicine at Mount Sinai, University of California San Diego, and Dana Farber Cancer Institute (DFCI protocol 16-273). Eligible HIV+ individuals were aged 40 or older and receiving 3 or more ART drugs for at least 2 years, including the last year prior to death, with the last plasma viral load (VL) within 12 months prior to death undetectable (<50 copies/mL) or <200 HIV RNA copies/mL and post-mortem interval (PMI) less than 24 h. Cases with active CNS infections or non-HAND neurodegenerative diseases were excluded. The following two cases not meeting all eligibility criteria were included: one case maintained on 2 ART drugs (AZT and 3TC; combivir) with plasma VL suppressed below 50 copies/mL for more than 3 years, and one case with PMI of 28 h. NCI status was determined based on HAND diagnoses and neurocognitive T-scores [41,42,43]. Three subjects with diagnoses of neuropsychological impairment due to other causes (NPI-O) were assigned to the unimpaired group because review of available clinical data suggested low probability of HAND based on neurocognitive T-scores in the normal range and/or confounding by heavy alcohol use. Brains were removed and examined at the individual NNTC sites [40], tissue was frozen or formalin-fixed, and gross pathology and histopathology evaluated by neuropathologists at each site. Features evaluated included inflammation, reactive astrocytes, macrophages/microglia, microglial nodules, and decreased myelin. HIVE scores were defined as: 0 = no or minimal inflammation, 1 = mild to moderate lymphocytic inflammation, 2 = microglial nodules, severe lymphocytic inflammation, and/or leukoencephalopathy [11,26].

### 2.2. Nucleic Acid Extraction and Brain HIV IPDA and Viral Load Measurements

Total RNA and genomic DNA were extracted from ~30 mg frozen frontal lobe white matter using the AllPrep DNA/RNA isolation kit according to the manufacturer’s instructions (Qiagen, Valencia, CA, USA). The IPDA assay was performed by Accelevir Diagnostics (Baltimore, MD, USA) using 8–12 µg genomic DNA isolated from homogenized frontal white matter as described [32,36,37,38,39]. As reported in Bruner et al. [32], the IPDA consists of two multiplex ddPCR reactions: the HIV proviral DNA reaction, which distinguishes intact from defective proviruses, and a second reaction performed in parallel that quantifies DNA shearing and input diploid cell equivalents. A DNA shearing correction is used to account for shearing using a DNA shearing index (DSI), and final IPDA results are reported as intact, defective, or total proviral genome frequencies per million brain cells. Paired samples of total RNA and genomic DNA from an adjacent region of the same tissue sample were assayed for HIV gag DNA/RNA levels by the HIV Molecular Monitoring Core (Frederick National Laboratory for Cancer Research, Frederick, MD, USA) using single-copy gag assays [11]. Input nucleic acid from 1 to 2 million cell equivalents (0.5–2 µg) was assayed in 12 replicates for each RNA and DNA sample. DNA cell equivalent-recoveries were assessed using an hCCR5 assay, which was used to normalize cellular HIV RNA and DNA viral loads.

### 2.3. Gene Expression Profiling

Targeted gene expression profiling was performed using NanoString nCounter technology (NanoString Technologies, Seattle, WA, USA) at the Dana-Farber/Harvard Cancer Center Molecular Biology core facility as described [11,26]. RNA content and quality (RIN; RNA integrity number) were evaluated using the Agilent BioAnalyzer (Agilent Technologies, Santa Clara, CA, USA). The probe set consisted of 78 probes to detect expression of genes related to inflammation, interferon responses, stress response, myeloid cells, T-cells, energy metabolism, and white matter integrity that we previously mapped to differentially expressed genes and co-expression modules in white matter from HIV+ vs. HIV− individuals [11]. Quality control checks were performed using nSolver 3.0 software (NanoString Technologies, Seattle, WA, USA).

### 2.4. Data Analysis

Group differences were evaluated by Wilcoxon rank-sum or Fisher’s exact test for continuous and categorical variables, respectively (*p*-values < 0.05). Spearman’s correlation analyses were performed in R version 4.2.1 (*p*-values < 0.05). Fold-change (FC) analysis of gene expression was calculated using Welch’s t-test (*p* < 0.05) and controlling for false-discovery rates (FDR < 0.10) estimated by the Benjamini–Hochberg method using the R package *fdrtool* (*version 1.2.17*).

## 3. Results

### 3.1. Characteristics of the Study Cohort

A total of 28 HIV+ individuals who were virally suppressed (<200 copies/mL) on ART within 12 months prior to death were selected from an autopsy cohort of 34 HIV+ individuals previously characterized for frontal white matter viral load and gene expression profiles in Solomon et al. [11,26]. Demographics, clinical characteristics, and laboratory data are summarized in Table 1. HIV+ individuals had an overall median age of 56 (IQR 50–60) years, 79% were male, median duration of HIV infection was 17 (IQR 13–22) years, and median last CD4 count was 288 (IQR 127–437) cells/μL. The last pre-mortem ART regimen included a protease inhibitor, non-nucleoside reverse transcriptase inhibitor, or integrase inhibitor in 17 (61%), 7 (25%), and 3 (11%) individuals, respectively.

Neurocognitive evaluations identified 15 HIV+ individuals with NCI, including 4 with HAD, 2 with MND, and 9 with NPI-O, while the remaining 13 were classified as unimpaired. NCI and non-NCI groups were similar in age (median 56 and 52 years, respectively), gender (67% and 92% male), race (33% and 54% white), duration of HIV infection (median 17 and 19 years) and CD4 counts (median 283 and 309 cells/μL), but NCI individuals had lower neurocognitive T-scores than non-NCI individuals (median 38 vs. 49, *p* = 0.0002) (Table 1). Although NCI and non-NCI groups had similar last premortem plasma and CSF VL within 12 or 18 months prior to death, respectively (median 40 copies/mL for both, corresponding to the lower limit of detection), there was a statistically significant difference or increasing trend between these groups (*p* = 0.01 and *p* = 0.11, respectively).

Given the significant association between NCI status and HIVE scores (*p* = 0.003), we also evaluated cohort characteristics by HIVE score (Supplemental Table S1). The HIVE score identified seven individuals with HIVE score = 1 and 2 with HIVE score = 2, while the remaining 19 HIV+ individuals had HIVE score = 0. No cases had classic HIVE with multinucleated giant cells and only one case had microglial nodule encephalitis. HIVE score 1 or 2 and HIVE score 0 groups were similar in age, gender, race, duration of HIV infection, CD4 counts, and CSF VL, but individuals with HIVE score 1 or 2 had an increasing trend for plasma VL and significantly lower neurocognitive T-scores than those with HIVE score 0 (*p* = 0.08 and *p* = 0.007, respectively).

### 3.2. IPDA Analysis and HIV DNA and RNA Brain Viral Load

**Figure 1:**
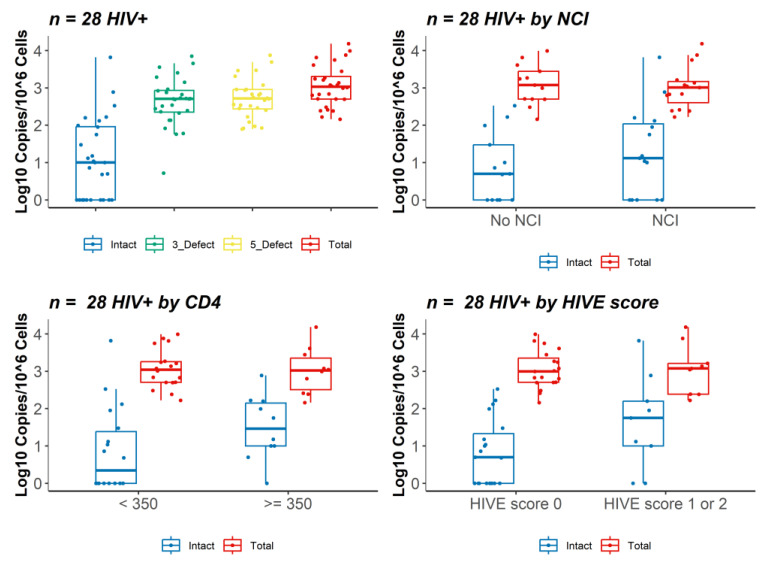
IPDA analysis of HIV proviruses in brain tissue from 28 HIV+ individuals on ART. Log10 copy number (standardized to 10^6^ cells) of proviral genomes defined as intact (blue), 3′ defective (green), 5′ defective (yellow), or total (sum of intact, 3′ defective, and 5′ defective; red) was measured in autopsy brain tissue (frontal white matter) from HIV+ individuals on ART (*n* = 28) (top left). Log10 copy number of intact and total proviruses in groups by neurocognitive impairment (NCI, *n* = 15) vs. no neurocognitive impairment (No NCI, *n* = 13), last CD4 count <350 (*n* = 18) vs. last CD4 count ≥350 (*n* = 10), or HIVE score 0 (*n* = 19) vs. HIVE score 1 or 2 (*n* = 9) (top right and bottom panels). Horizontal bars represent medians, boxes span the interquartile range (IQR), and whiskers extend to extreme data points within 1.5 times the IQR.

IPDA analysis was performed on DNA isolated from frozen brain tissue (frontal white matter) of 28 HIV+ individuals. We selected the frontal lobe because this region is commonly used in studies that analyze brain viral load [1,7,10,11,16,17,18]. Single-copy HIV DNA and RNA VL assays detecting an HIV gag target were performed on DNA/RNA isolated from an adjacent region of the same tissue sample [1]. A summary of the intact, defective, and total proviral copy number measured by IPDA and HIV gag DNA and RNA VL in brain tissue of 28 HIV+ individuals is shown in Table 1 and Figure 1. The median proviral genome copy numbers per 10^6^ cells in brain tissue as measured by IPDA were as follows: intact, 10 (IQR 1–92); 3′ defective, 509 (IQR 225–858); 5′ defective, 519 (IQR 273–906); and total proviruses, 1063 (501–2074) copies/10^6^ cells. Intact proviral genomes were detected at >1 copy per 10^6^ cells in frontal white matter in 18 of 28 individuals (64%), while 3′ defective and 5′ defective proviral genomes were detected in all 28 individuals. Total proviral genome value was calculated by summing intact, 3′ defective, and 5′ defective proviral genomes; intact proviral genomes accounted for less than 10% (mean 1.8%, median 8.3%; *n* = 28) of total proviral genomes in the brain of most individuals, while 3′ and 5′ defective genomes accounted for 44% and 49%, respectively. Median HIV gag DNA was 8.7 (IQR 4.7–13.1) copies/10^6^ cells and HIV gag RNA was 7.5 (IQR 1.5–27.9) copies/10^6^ cells.

To evaluate relationships between intact, defective, or total proviruses and neurocognitive or neuroinflammation status, we compared results from IPDA and viral load analysis between groups stratified by NCI or HIVE score (Figure 1, Table 1, and Supplemental Table S1). There was no significant difference in median copy number of intact proviruses (13 vs. 5 copies/10^6^ cells, *p* = 0.4), 3′ defective proviruses (489 vs. 531 copies/10^6^ cells, *p* = 0.3), 5′ defective proviruses (509 vs. 645 copies/10^6^ cells, *p* = 0.4), or total proviruses (1024 vs. 1181 copies/10^6^ cells, *p* = 0.7) between groups stratified by NCI vs. no NCI, respectively. Brain HIV gag DNA trended toward higher levels in NCI (9.7 vs. 5.0 copies/10^6^ cells, *p* = 0.05), but HIV gag RNA was not significantly different (7.0 vs. 7.9 cps/10^6^ cells, *p* = 0.9) between NCI vs. non-NCI groups. A summary of proviral copy numbers measured by IPDA and HIV DNA and RNA VL values among the 28 HIV+ individuals stratified by HIVE score is shown in Supplemental Table S1. There was an increasing trend in intact proviruses (56 vs. 5 copies/10^6^ cells, *p* = 0.1) in HIVE score 1 or 2 (*n* = 9) vs. HIVE score 0 (*n* = 19) groups, but no significant differences in 3′ defective proviruses (508 vs. 516 copies/10^6^ cells, *p* = 0.5), 5′ defective proviruses (559 vs. 469 copies/10^6^ cells, *p* = 0.7), or total proviruses (1189 vs. 991 copies/10^6^ cells, *p* = 0.9) (Supplemental Table S1). Brain HIV gag DNA VL was higher in HIVE score 1 or 2 vs. HIVE score 0 groups (median 13.0 vs. 7.1 copies/10^6^ cells, *p* = 0.02), while there was no difference in brain HIV gag RNA VL (2.8 vs. 12.0 copies/10^6^ cells, *p* = 0.6). We also evaluated groups by last CD4 count <350 (*n* = 18) vs. ≥ 350 (*n* = 10). Intact proviruses showed a trend toward higher levels in the CD4 count ≥350 vs. <350 group (median 35 vs. 3 copies/10^6^ cells, respectively, *p* = 0.06), while total proviruses were similar (median 1102 vs. 1047 copies/10^6^ cells, respectively, *p* = 0.7) (Figure 1). These results suggest that neuroinflammation but not NCI is associated with higher levels of intact proviruses in HIV+ individuals on suppressive ART.

### 3.3. Correlation Analysis of Intact Proviruses versus Total Proviruses or Gag DNA Levels

**Figure 2:**
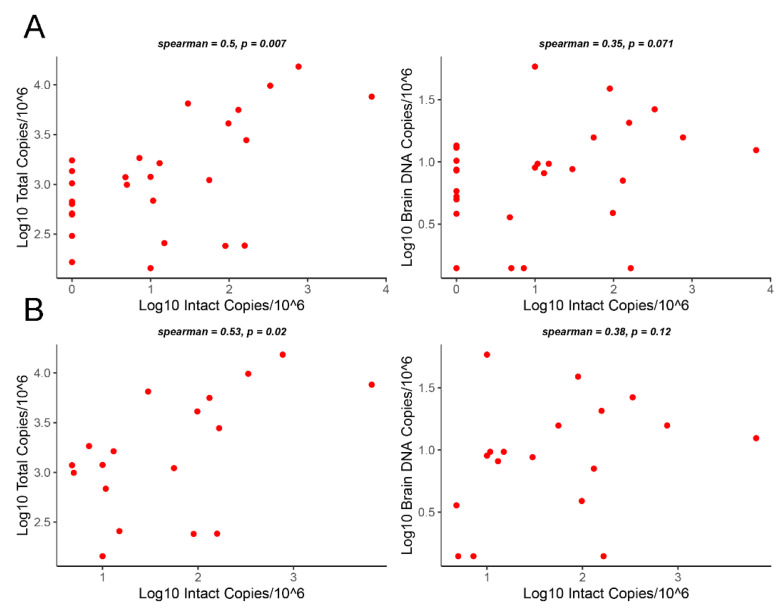
Correlation analysis of intact versus total HIV provirus or gag DNA levels in brain tissue from HIV+ individuals on ART. (**A**) Correlation between log10 intact provirus copy number and log10 total provirus copy number or log10 gag DNA copy number (standardized to 10^6^ cells) in brain tissue (frontal lobe white matter) among 28 HIV+ individuals. (**B**) Correlation analysis as described in sensitivity analysis (**A**) restricted to 18 HIV+ individuals with detectable intact proviruses (>1 intact copy per 10^6^ cells). Spearman’s rho and *p*-values are shown.

Next, we used Spearman’s rank correlation to assess the relationship between log10 intact proviruses and log10 total proviruses or log10 gag DNA in brain tissue among all 28 HIV+ individuals (Figure 2A), or the 18 HIV+ individuals with detectable intact provirus copies (>1 intact copy per 10^6^ cells) (Figure 2B). There was a positive correlation between log10 intact provirus and log10 total provirus copy number (r = 0.5, *p* = 0.007 and r = 0.53, *p* = 0.02 for *n* = 28 and *n* = 18 HIV+ individuals, respectively). We also observed a positive correlation between the log10 intact provirus copy number and log10 gag DNA copy number (r = 0.35, *p* = 0.071 and r = 0.38, *p* = 0.12 for *n* = 28 and *n* = 18 HIV+ individuals, respectively), but not log10 RNA copy number (r = 0.19, *p* = 0.93). These findings suggest that intact proviruses are predictive of total proviruses in brain tissue from HIV+ individuals on suppressive ART. In contrast, there was only a weak correlation between log10 intact proviruses and HIV gag DNA levels and no significant correlation between log10 intact proviruses and HIV gag RNA levels in brain tissue.

### 3.4. Gene Expression Profiles Stratified by Level of Intact Proviruses in Brain Tissue

We compared white matter gene expression profiles between groups stratified by intact proviruses >5 copies vs. <=5 copies per 10^6^ cells in brain tissue from the 28 HIV+ individuals on ART. Differential expression analysis was conducted on a target set of 78 genes mapping to 7 co-expression modules including differentially expressed genes that we previously identified in brain tissue from 34 HIV+ individuals on ART vs. 24 HIV- controls using the NanoString platform [11,26]. These co-expression modules represent seven functional categories: inflammation/interferon response, stress response, myeloid cells, MHC-1, T-cells, oligodendrocytes, and oxidative phosphorylation/energy metabolism.

First, we compared expression of these 78 genes between HIV+ (*n* = 28) and HIV− (*n* = 20) individuals matched for age and sex. Most of these genes (68/78) showed increased expression in white matter from HIV+ individuals compared to HIV- controls, while several oxidative phosphorylation/energy metabolism (PARK2, SREBF1, SREBF2, TXNIP) and oligodendrocyte (OLIG1, MAG, MOG) genes showed decreased expression (Supplemental Table S2). Among the 78 genes, 44 genes were differentially expressed (*p* < 0.05 and FDR < 0.10), and 10 genes showed modest differences (*p* < 0.1 and FDR < 0.15) between HIV+ vs. HIV- individuals. As expected, results for most of these differentially expressed genes were consistent with expression profiles in Solomon et al. [11,26].

**Figure 3:**
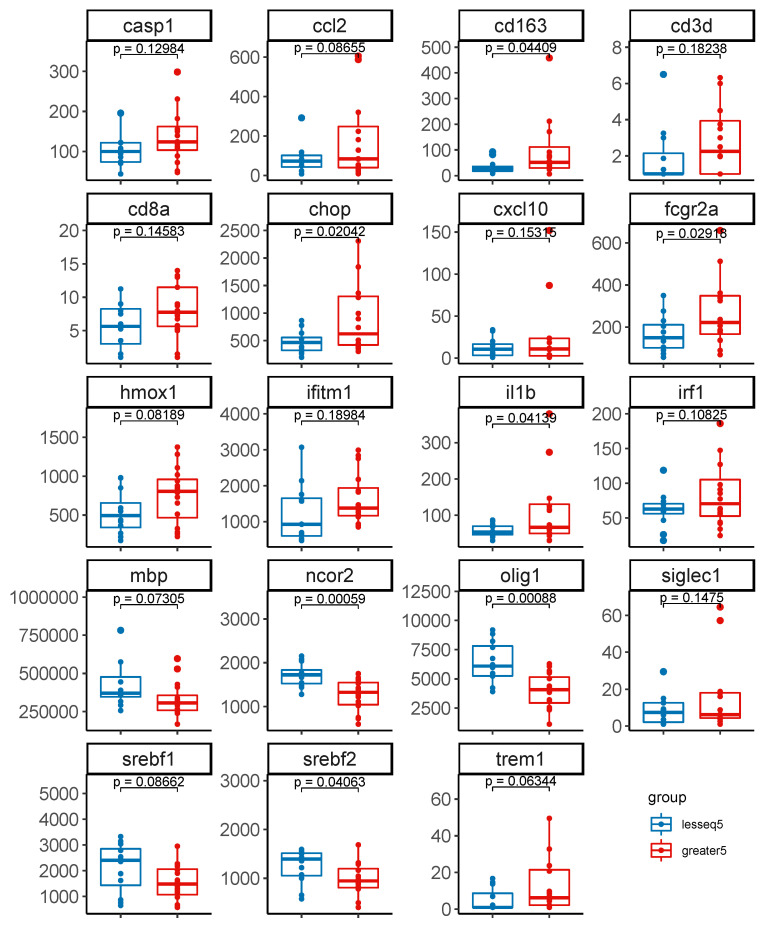
Differential expression of genes related to neuroinflammation and white matter integrity in groups stratified by level of intact proviruses in brain tissue from 28 HIV+ individuals on ART. Differential gene expression in brain tissue (frontal white matter) from 28 HIV+ individuals stratified by frequency of intact proviruses per 10^6^ cells in brain tissue >5 copies vs. ≤5 copies per 10^6^ cells (*n* = 12 and *n* = 16, respectively). Horizontal bars represent medians, boxes span the interquartile range (IQR), and whiskers extend to extreme data points within 1.5 times the IQR. *p*-values calculated using Welch’s t-test.

We further analyzed differential expression of these 78 genes in groups stratified by level of intact proviruses in brain tissue >5 copies vs. ≤5 copies per 10^6^ cells (*n* = 16 and *n* = 12, respectively). A total of 19 of 78 genes met non-stringent criteria of FC > 1.3 and unadjusted *p*-value < 0.2 (Supplemental Table S2). Differential expression of these 19 genes between groups stratified by level of intact proviruses per 10^6^ cells in brain tissue is shown in Figure 3. These genes are related to inflammation and interferon responses (*n* = 5; CXCL10, IFITM1, SIGLEC1, IRF1, FCGR2A), stress responses (*n* = 4; CASP1, CHOP, IL1B, HMOX1), T-cells (*n* = 2; CD3D, CD8A), oligodendrocytes (*n* = 2; MBP, OLIG1), myeloid cells (*n* = 3; CCL2, CD163, TREM1), and oxidative phosphorylation/energy metabolism (*n* = 3; NCOR2, SREBF1, SREBF2). Among the 19 genes, 7 genes (CD163, CHOP, FCGR2A, IL1B, NCOR2, OLIG1, SREBF2) were significantly different (*p* < 0.05), 5 genes (CCL2, HMOX1, MBP, SREBF1, TREM) showed modest differences (*p* < 0.1), and 7 genes (CASP1, CD3D, CD8A, CXCL10, IFITM1, IRF1, SIGLEC1) showed trends for differential expression between groups stratified by intact proviruses >5 vs. ≤5 copies per 10^6^ cells (*p* < 0.2). The majority of differentially expressed genes (14/19) showed modest increases in expression in white matter with >5 vs. ≤5 intact provirus copies per 10^6^ cells. In contrast, expression of energy- and lipid metabolism-related genes (SREBF1, SREBF2) and oligodendrocyte-associated genes (OLIG1, MBP) showed modest decreases in expression in white matter with >5 vs. ≤5 intact provirus copies per 10^6^ cells. These results suggest an association between intact proviruses and changes in expression of genes related to neuroinflammation, stress responses, and white matter integrity.

Given modest differences in the preceding analyses, we further analyzed white matter gene expression profiles of the two groups stratified by level of intact proviruses in comparison to a group of HIV- individuals. Supplementary Figure S1 shows differential expression of the 19 genes related to neuroinflammation and white matter integrity in brain tissue from the 28 HIV+ individuals stratified by intact proviruses per 10^6^ cells >5 vs. ≤5 copies (*n* = 16 and *n* = 12, respectively) in comparison to 20 HIV- controls. Most of these genes (12/19) showed increased expression in HIV+ individuals in both groups compared to HIV- individuals. In contrast, expression of energy metabolism-related genes (SREBF1, SREBF2) and oligodendrocyte-associated genes (OLIG1, MBP) was higher in HIV- controls compared to HIV+ individuals with intact proviruses >5 copies per 10^6^ cells, albeit lower than HIV+ individuals with intact proviruses ≤5 copies per 10^6^ cells. Most of these genes (13/19) showed significant differences (*p* < 0.05) between HIV+ individuals with intact proviruses >5 copies per 10^6^ cells vs. HIV- individuals, while fewer genes (4/19) had significant differences between HIV+ individuals with intact proviruses ≤5 copies per 10^6^ cells vs. HIV- individuals.

## 4. Discussion

This is the largest study to date using the IPDA to measure intact, defective, and total HIV proviral genomes in brain tissue from virally suppressed HIV+ individuals on ART. Intact proviral genomes were detected in frontal white matter in the majority (64%) of HIV+ individuals on suppressive ART, with a median intact proviral genome copy number of 10 copies/10^6^ cells (IQR 1–92). These intact proviral genome copy numbers in brain tissue are in a similar range to those previously reported in blood and lymphoid tissues [32,36,37,38,39]. Our findings are consistent with Cochrane et al. [18], who reported intact proviral genome copy numbers of 4.6 and 12.7 intact copies/10^6^ cells in brain tissue from viremic and virally suppressed individuals, respectively. We found that the level of intact proviral genomes correlated positively with total proviral genomes and HIV gag DNA, but not HIV gag RNA in brain tissue, most likely because intact proviral genomes represent both latent and replicating viruses. Intact proviral genomes accounted for less than 10% (median 8.3%) of total proviral genomes in brain of most individuals. As reported by others [18,32,36,39], most proviruses were 3′ or 5′ defective proviruses, accounting for ~44% and 49% of total proviral genomes, respectively. This finding has potential biological significance because defective proviruses are still capable of producing viral proteins such as Tat, Nef, and Env, which are potentially neurotoxic [2,22] and may therefore contribute to HAND in some virally suppressed individuals. However, the regulation of viral transcriptional activity in the brains of HIV+ individuals on ART is multifactorial and complex, which may partially explain why most of our findings did not correlate directly with NCI [2].

Although we detected slightly higher HIV gag DNA levels in HIV+ individuals with NCI vs. no NCI, there was no significant difference in median copy number of intact, defective, or total proviruses or HIV RNA levels between groups stratified by NCI vs. no NCI. In contrast, HIVE score 1 or 2 (predominantly mild-to-moderate lymphocytic inflammation) was associated with higher gag DNA levels (*p* = 0.02) and increasing trend for intact proviruses (*p* = 0.1), but no significant difference in the level of defective or total proviruses. Thus, intact proviral genomes were more closely related to neuroinflammation than NCI status in virally suppressed individuals on ART. Furthermore, 5 of 6 cases with myelin pallor vs. 11 of 22 cases without myelin pallor had >5 intact proviruses/10^6^ cells in frontal white matter (83% vs. 50%; Fisher’s exact test *p* = 0.14). While classic HIVE with multinucleated giant cells, microglial nodules, and myelin loss was not seen in these virally suppressed cases, microgliosis, lymphocytic infiltration, and white matter abnormalities can contribute to NCI [11,13,14,16,23,26,27,44]. These findings, together with the observation that neuroinflammatory pathology was more frequent among individuals with NCI (*p* = 0.003), suggest that presence of replication-competent HIV and associated CNS inflammation/immune activation are likely factors contributing to an inflammatory subtype of HAND in some individuals on suppressive ART [12,13,14,45,46].

Genes related to inflammation, stress responses, and white matter integrity were differentially expressed in brain tissues with >5 vs. ≤5 intact proviruses/10^6^ cells. These genes are associated with inflammation and interferon responses (CXCL10, IFITM1, SIGLEC1, IRF1, FCGR2A), stress responses (CASP1, CHOP, IL1B, HMOX1), T-cells (CD3D, CD8A), myelin/oligodendrocytes (MBP, OLIG1), myeloid cells (CCL2, CD163, TREM1), and lipid/energy metabolism (NCOR2, SREBF1, SREBF2). Consistent with prior studies [11,26,28,29,30,47,48], genes related to inflammation/interferon response, stress response, myeloid cells, and T-cells were upregulated in HIV+ individuals vs. HIV- controls, while genes related to white matter/oligodendrocytes were downregulated. Many of these gene expression changes were augmented in brain tissues with >5 vs. ≤5 intact proviruses/10^6^ cells when compared with HIV- controls. Our finding that expression of genes related to inflammation and macrophage/microglial activation was higher in brain tissues with >5 intact proviruses/10^6^ cells is consistent with previous studies indicating that low-level HIV infection of the CNS induces neuroinflammation and myeloid cell activation [1,11,12,16,27,31,48]. Downregulation of lipid/cholesterol metabolism-related genes in brain tissues with >5 vs. ≤5 intact proviruses/10^6^ cells is relevant for the white matter abnormalities frequently observed in aviremic HIV+ individuals [16,23,44] because these genes are important for myelination [49,50,51].

Limitations of this study include heterogeneity of the study cohort, including timing of ART initiation, duration of viral suppression on ART, type of ART regimen, subset with NPI-O, and comorbidities. Additionally, there were gaps in time between the last plasma VL and death; some individuals may have stopped taking ART drugs near the time of death. Moreover, we did not measure ART drug concentrations in brain tissue samples. HIV+ individuals had a median duration of HIV infection of 17 years, and autopsies were performed between 2001 and 2014. As such, these individuals received many different ART regimens; the majority were on protease inhibitors and a minority on integrase inhibitors at the last visit prior to death. Although all participants had multiple plasma VL measurements in the years before death, only 14 had one or more CSF VL. For brain IPDA, HIV gag DNA/RNA assays, and gene expression profiling, only a single anatomical site (frontal white matter) was tested for each subject, which can lead to sampling bias. Mismatches between HIV sequences in target sequences and primers used for amplification can also lead to detection bias. NanoString is a multiplex approach to digital counting of mRNAs, but has a lower dynamic range compared to RNAseq. Due to the heterogeneous cell types in brain tissue, we could not attribute changes specifically to any particular cell type. The gene expression profiles were only indicative, as we did not have sufficient statistical power to detect modest differences in expression among 78 genes. Future studies using single-cell technologies and contemporary cohorts are needed to further define cellular reservoirs of HIV in the brain and associated gene expression changes [31,52,53,54].

## 5. Conclusions

This study used the IPDA to evaluate the brain viral reservoir in virally suppressed HIV+ individuals and evaluate relationships between intact proviruses and NCI, neuroinflammatory pathology, and gene expression profiles. Our findings suggest that intact proviral genomes are present in the brain in the majority of HIV+ individuals at levels comparable to those reported in blood and lymphoid tissues, despite suppressive ART. We further demonstrate that intact proviral genome levels are more closely related to neuroinflammation than NCI status and associate with differential expression of genes related to neuroinflammation and white matter integrity in virally suppressed individuals. These findings highlight the importance of targeting the CNS viral reservoir to achieve an HIV cure. Further studies of larger cohorts on contemporary ART regimens and using single-cell technologies to evaluate viral reservoirs and associated gene expression changes will be important to gain better mechanistic understanding of the relationship between replication-competent proviruses and inflammatory pathologies in the brain and other anatomical sites.

## Figures and Tables

**Table 1:** Clinical and virological characteristics of the study cohort by neurocognitive status.

Variable	Total (*n* = 28)	No NCI (*n* = 13)	NCI (*n* = 15)	*p*-Value
Age (years)	55.50 (50.00, 59.50)	52.00 (50.00, 59.00)	56.00 (52.00, 60.00)	0.5182
Male gender, *n* (%)	22 (78.6)	12 (92.3)	10 (66.7)	0.2351
Race/ethnicity, *n* (%)				0.5685
White	12 (42.9)	7 (53.8)	5 (33.3)	
Black	11 (39.3)	4 (30.8)	7 (46.7)	
Hispanic	4 (14.3)	2 (15.4)	2 (13.3)	
Other	1 (3.6)	0 (0.0)	1 (6.7)	
PMI (hours)	12.00 (5.88, 16.25)	12.00 (6.00, 15.00)	11.00 (6.75, 18.25)	0.9082
Duration of HIV infection (years)	17.00 (13.00, 21.50)	18.50 (10.00, 21.25)	17.00 (14.00, 20.75)	0.7322
CD4 count (cells/μL)	287.50 (126.25, 436.50)	309.00 (128.00, 427.00)	283.00 (126.50, 454.00)	0.8719
CD4 nadir (cells/μL)	98.00 (53.50, 147.50)	78.00 (17.00, 128.00)	98.00 (65.00, 185.00)	0.4201
Plasma VL (copies/mL) *	40.00 (40.00, 40.00)	40.00 (40.00, 40.00)	40.00 (40.00, 77.00)	0.0123
Time between last plasma VL and death (years)	0.40 (0.20, 0.62)	0.40 (0.20, 0.50)	0.40 (0.15, 0.65)	0.7801
CSF VL (copies/mL) *	40.00 (40.00, 40.00)	40.00 (40.00, 40.00)	40.00 (40.00, 137.50)	0.1119
Time between last CSF VL and death (years)	0.30 (0.00, 1.00)	0.20 (0.00, 0.75)	0.35 (0.07, 1.23)	0.6065
Maximum CSF VL in study (copies/mL)	40.00 (40.00, 170.00)	40.00 (40.00, 77,174.00)	40.00 (40.00, 40.00)	0.3102
Neurocognitive T-score	43.80 (37.27, 48.69)	49.14 (47.32, 53.24)	38.08 (36.16, 43.30)	0.0002
HAND diagnosis, *n* (%)				0.0005
MND	2 (7.4)	0 (0.0)	2 (13.3)	
HAD	4 (14.8)	0 (0.0)	4 (26.7)	
NPI-O	12 (44.4)	3 (25.0)	9 (60.0)	
Normal	9 (33.3)	9 (75.0)	0 (0.0)	
HIVE score, *n* (%)				0.0032
0	19 (67.9)	13 (100.0)	6 (40.0)	
1	7 (25.0)	0 (0.0)	7 (46.7)	
2	2 (7.1)	0 (0.0)	2 (13.3)	
Intact proviruses (cps/10^6^ cells)	10.00 (1.00, 91.96)	5.00 (1.00, 30.00)	13.05 (1.00, 110.74)	0.3831
3′ defective proviruses (cps/10^6^ cells)	508.71 (224.80, 857.78)	530.92 (229.15, 1422.37)	488.60 (205.88, 590.31)	0.3001
5′ defective proviruses (cps/10^6^ cells)	519.09 (273.18, 905.71)	645.03 (280.45, 1191.33)	508.49 (226.47, 686.78)	0.4472
Total proviruses (cps/10^6^ cells)	1063.93 (501.28, 2074.33)	1180.74 (502.72, 2779.76)	1024.07 (445.79, 1495.22)	0.6617
HIV gag DNA (cps/10^6^ cells)	8.71 (4.72, 13.13)	5.00 (3.59, 8.75)	9.67 (8.40, 14.36)	0.0527
HIV gag RNA (cps/10^6^ cells)	7.45 (1.51, 27.92)	7.92 (1.12, 25.28)	6.98 (2.32, 31.08)	0.8755

All data are median [interquartile range] unless otherwise indicated. *p* values for two group comparisons were calculated using Fisher’s exact test for categorical variables or Wilcoxon rank sum test for continuous variables. CSF VL within 18 months prior to death was not available for 15 individuals. HAND diagnosis was not available for 1 individual. * 22/28 (79%) and 11/13 (85%) were undetectable at <=40 copies/ml in plasma and CSF, respectively; all individuals with detectable values (41–200 copies/ml) were in the NCI group. Abbreviations: ART, antiretroviral therapy; HAD, HIV-associated dementia; HAND, HIV-associated neurocognitive disorder; HIVE, HIV encephalitis; MND, mild neurocognitive disorder; NCI, neurocognitive impairment; NPI-O, neuropsychological impairment attributable to other causes; PMI, post-mortem interval; VL, viral load.

## Data Availability

All data generated or analyzed during this study are included in the published article and its Supplementary Information files or available from the corresponding author on reasonable request.

## Supplementary Materials

The following supporting information can be downloaded at: https://www.mdpi.com/article/10.3390/v15041009/s1, Figure S1: Differential expression of genes related to neuroinflammation and white matter integrity in brain tissue from 28 HIV+ individuals on ART groups stratified by HIV status and level of intact proviruses; Table S1: Clinical and virological characteristics of the study cohort by HIVE score; Table S2: Differential expression analysis of 78 genes mapping to interferon response, stress response, oxidative phosphorylation, MHC-I upregulation, and cell-type marker clusters.
