# Supplementary material for: Intact Proviral DNA Analysis of the Brain Viral Reservoir and Relationship to Neuroinflammation in People with HIV on Suppressive Antiretroviral Therapy
Source: Viruses. 2023 Apr 20;15(4):1009. doi: 10.3390/v15041009 (PMC10142371; doi:10.3390/v15041009)
Supplement: Supplementary file 1 [file viruses-15-01009-s001.zip › Supplemental Figure S1.pdf]

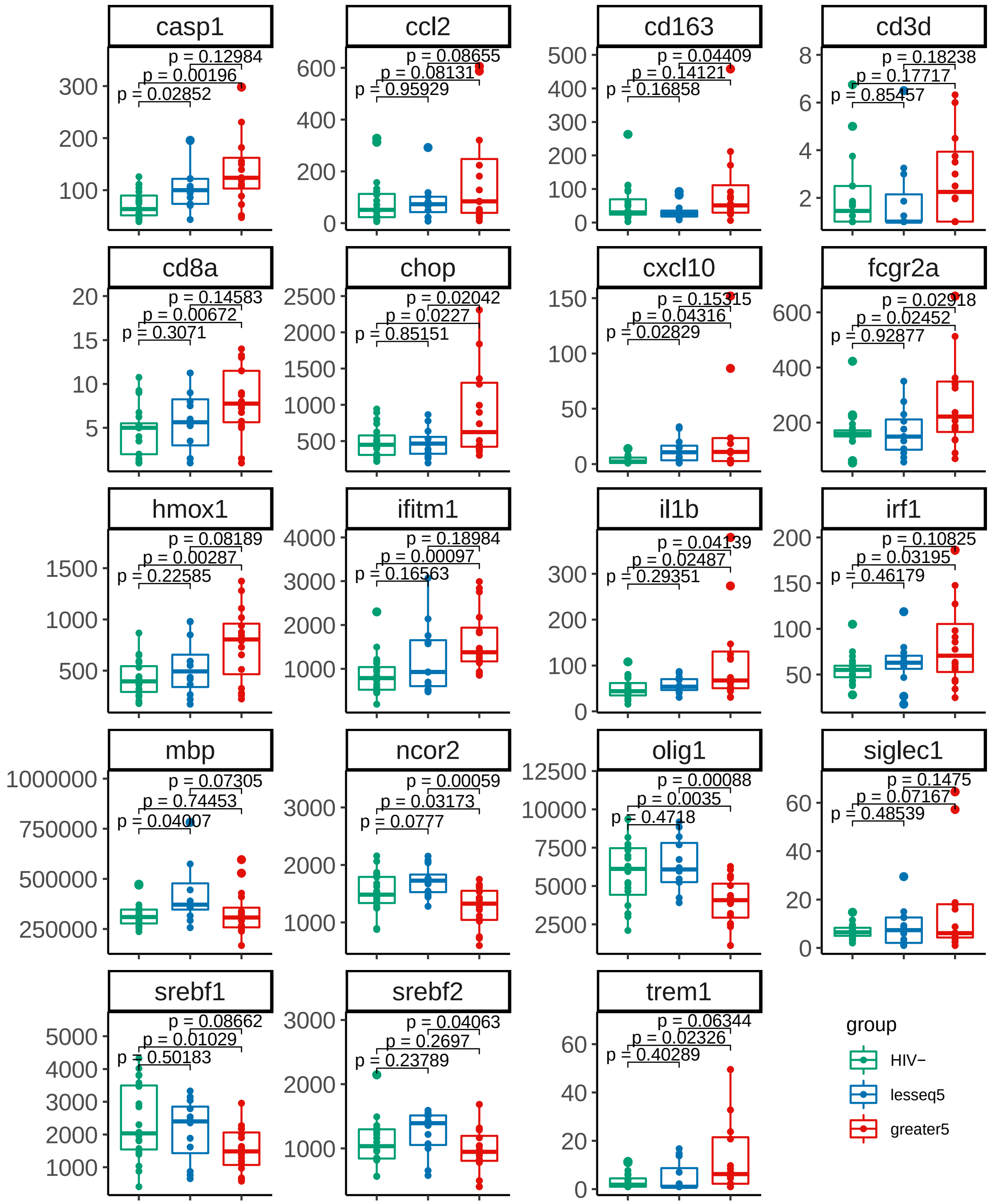

Supplemental Figure S1. Differential expression of genes related to neuroinflammation and white matter integrity in brain tissue from 28 HIV+ individuals on ART groups stratified by HIV status and level of intact proviruses. Horizontal bars represent medians, boxes span the interquartile range (IQR), and whiskers extend to extreme data points within 1.5 times the IQR. P values calculated using Welch's t test (n = 20 HIV-, n = 12 HIV+ with intact proviruses <= 5 copies per 10^6 cells, n = 16 HIV+ with intact proviruses >5 copies per 10^6 cells).
