# Supplementary material for: Intact Proviral DNA Analysis of the Brain Viral Reservoir and Relationship to Neuroinflammation in People with HIV on Suppressive Antiretroviral Therapy
Source: Viruses. 2023 Apr 20;15(4):1009. doi: 10.3390/v15041009 (PMC10142371; doi:10.3390/v15041009)
Supplement: Supplementary file 1 [file viruses-15-01009-s001.zip › Supplemental Table S1.pdf]

Supplemental Table 1. Clinical and virological characteristics of the study cohort by HIVE score.

Variable	All participants (n=28)	HIVE score = 0 (n=19)	HIVE score = 1 or 2 (n=9)	P value
Age (years)	55.50 [50.00, 59.50]	54.00 [51.00, 62.00]	56.00 [50.00, 59.00]	0.7301
Male gender, n (%)	22 (78.6)	16 (84.2)	6 (66.7)	0.5731
Race/ethnicity, n (%)				0.6987
White	12 (42.9)	9 (47.4)	3 (33.3)	
Black	11 (39.3)	7 (36.8)	4 (44.4)	
Hispanic	4 (14.3)	2 (10.5)	2 (22.2)	
Other	1 (3.6)	1 (5.3)	0 (0.0)	
PMI (hours)	12.00 [5.88, 16.25]	12.00 [5.75, 15.50]	12.00 [8.00, 18.50]	0.4599
Duration of HIV infection (years)	17.00 [13.00, 21.50]	19.25 [10.75, 21.75]	17.00 [15.00, 19.00]	0.8772
CD4 count (cells/μL)	287.50 [126.25, 436.50]	292.00 [124.50, 446.00]	283.00 [136.00, 421.00]	0.9021
CD4 nadir (cells/uL)	98.00 [53.50, 147.50]	98.00 [56.50, 162.00]	98.00 [54.00, 125.00]	0.7306
Plasma VL (copies/mL)	40.00 [40.00, 40.00]	40.00 [40.00, 40.00]	40.00 [40.00, 72.00]	0.0867
Time between last plasma VL and death (years)	0.40 [0.20, 0.62]	0.30 [0.20, 0.50]	0.40 [0.40, 0.70]	0.3326
CSF VL (copies/mL)	40.00 [40.00, 40.00]	40.00 [40.00, 40.00]	40.00 [40.00, 88.25]	0.4617
Time between last CSF VL and death (years)	0.30 [0.00, 1.00]	0.00 [0.00, 0.50]	0.95 [0.38, 1.62]	0.0675
Maximum CSF VL in study (copies/mL)	40.00 [40.00, 170.00]	40.00 [40.00, 170.00]	40.00 [40.00, 126.75]	0.7254
Neurocognitive T score	43.80 [37.27, 48.69]	46.50 [41.69, 50.14]	38.08 [28.92, 40.43]	0.0065
HAND diagnosis, n (%)				0.0411
MND	2 (7.4)	1 (5.6)	1 (11.1)	
HAD	4 (14.8)	1 (5.6)	3 (33.3)	
NPI-O	12 (44.4)	7 (38.9)	5 (55.6)	
Normal	9 (33.3)	9 (50.0)	0 (0.0)	
HIVE score, n (%)				<0.0001
0	19 (67.9)	19 (100.0)	0 (0.0)	
1	7 (25.0)	0 (0.0)	7 (77.8)	
2	2 (7.1)	0 (0.0)	2 (22.2)	
Intact proviruses (cps/10^6 cells)	10.00 [1.00, 91.96]	5.00 [1.00, 22.50]	55.97 [10.00, 158.22]	0.1071
3' defective proviruses (cps/10^6 cells)	508.71 [224.80, 857.78]	515.58 [237.38, 1,173.34]	508.05 [82.76, 665.04]	0.4757
5' defective proviruses (cps/10^6 cells)	519.09 [273.18, 905.71]	468.47 [285.71, 1,049.49]	559.22 [93.39, 694.41]	0.7122
Total proviruses (cps/10^6 cells)	1,063.93 [501.28, 2,074.33]	990.70 [506.16, 2,309.48]	1,188.52 [242.42, 1,631.00]	0.9412
HIV gag DNA (cps/10^6 cells)	8.71 [4.72, 13.13]	7.08 [3.71, 9.67]	13.00 [9.00, 15.73]	0.0193
HIV gag RNA (cps/10^6 cells)	7.45 [1.51, 27.92]	12.00 [2.80, 20.00]	2.80 [0.84, 45.24]	0.6064

All data are median [interquartile range] unless otherwise indicated. P values for two group comparisons were calculated using Fisher's exact test for categorical variables or Wilcoxon rank sum test for continuous variables. CSF VL within 18 months prior to death was not available for 15 individuals. HAND diagnosis was not available for 1 individual.

Abbreviations: ART, antiretroviral therapy; HAD, HIV-associated dementia; HAND, HIV-associated neurocognitive disorder; HIVE, HIV encephalitis; MND, mild neurocognitive disorder; NCI, neurocognitive impairment; NPI-O, neuropsychological impairment attributable to other causes; PMI, post-mortem interval; VL, viral load.
